# Newborn with amniotic band sequence

**DOI:** 10.1002/ccr3.7655

**Published:** 2023-08-22

**Authors:** Jesus Ruiz

**Affiliations:** ^1^ Department of Family Medicine University of North Carolina at Chapel Hill School of Medicine Chapel Hill North Carolina USA

**Keywords:** amniotic band sequence, amniotic band syndrome

## Abstract

**Key Clinical Message:**

Amniotic band sequence (ABS) should be on the differential for newborns with limb defects. ABS is diagnosable prenatally with prenatal ultrasound; however, there are cases where the diagnosis of ABS is made only after delivery of the newborn.

**Abstract:**

Amniotic band sequence (ABS) is an uncommon congenital disorder where strands of amniotic tissue cause entrapment of the limbs, body wall, and viscera leading to an array of congenital malformations. We report a case of a newborn with prenatally undiagnosed amniotic band sequence.

## Graphical abstract

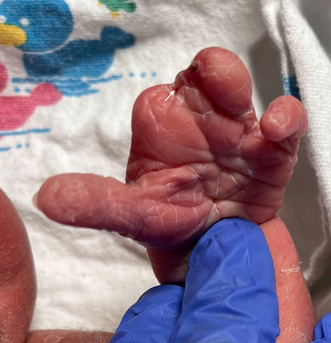

## 1 CASE PRESENTATION

A newborn baby girl was born to a 27‐year‐old female at 38 weeks and 6 days via normal spontaneous vaginal delivery. Pregnancy was complicated by late entry to prenatal care with a single prenatal ultrasound at 33 weeks of gestation without abnormalities; however, views were suboptimal and limited by gestational age. The patient's mother did not return for follow‐up imaging due to socioeconomic barriers. The patient's mother immigrated from Mexico at 28 weeks of pregnancy, and her first prenatal visit was at 30 weeks of gestation. The patient's mother had no medical conditions, no family history of congenital or genetic conditions, no known exposure to teratogens, and no history of substance use.

**FIGURE 1:**
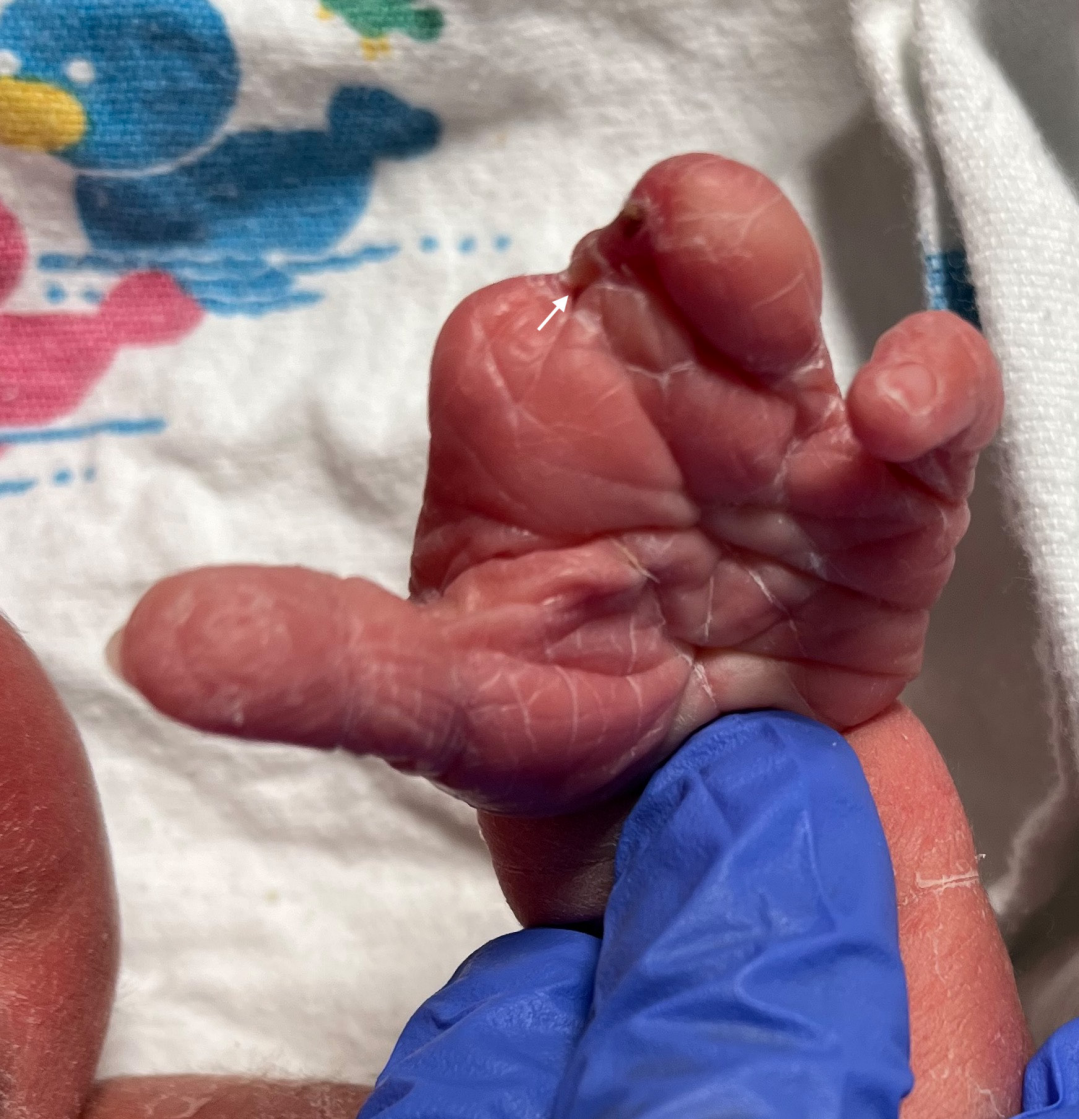
Truncation of the second through fourth digits of the left hand with connecting bands (arrow).

**FIGURE 2:**
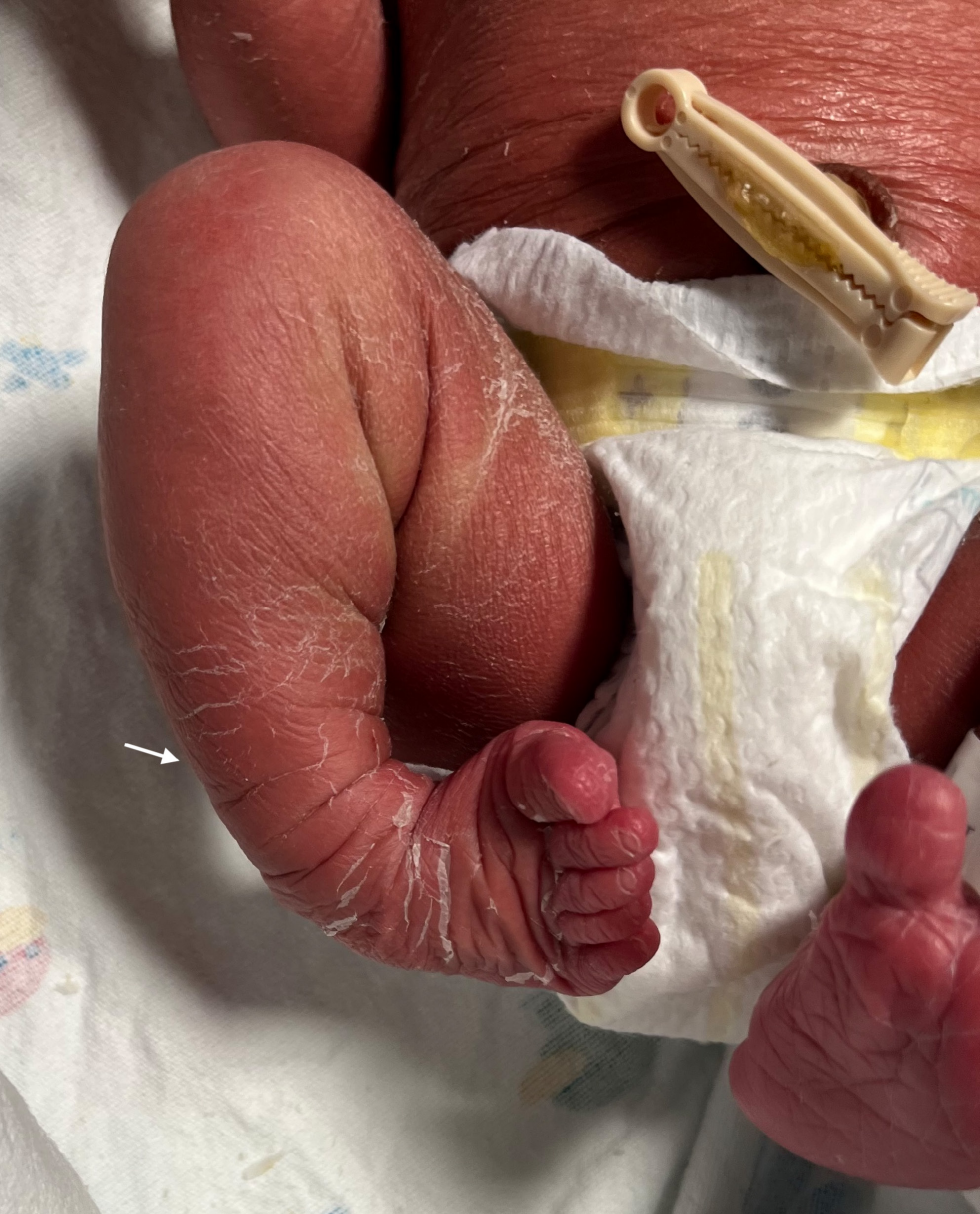
Talipes equinovarus (arrow).

**FIGURE 3:**
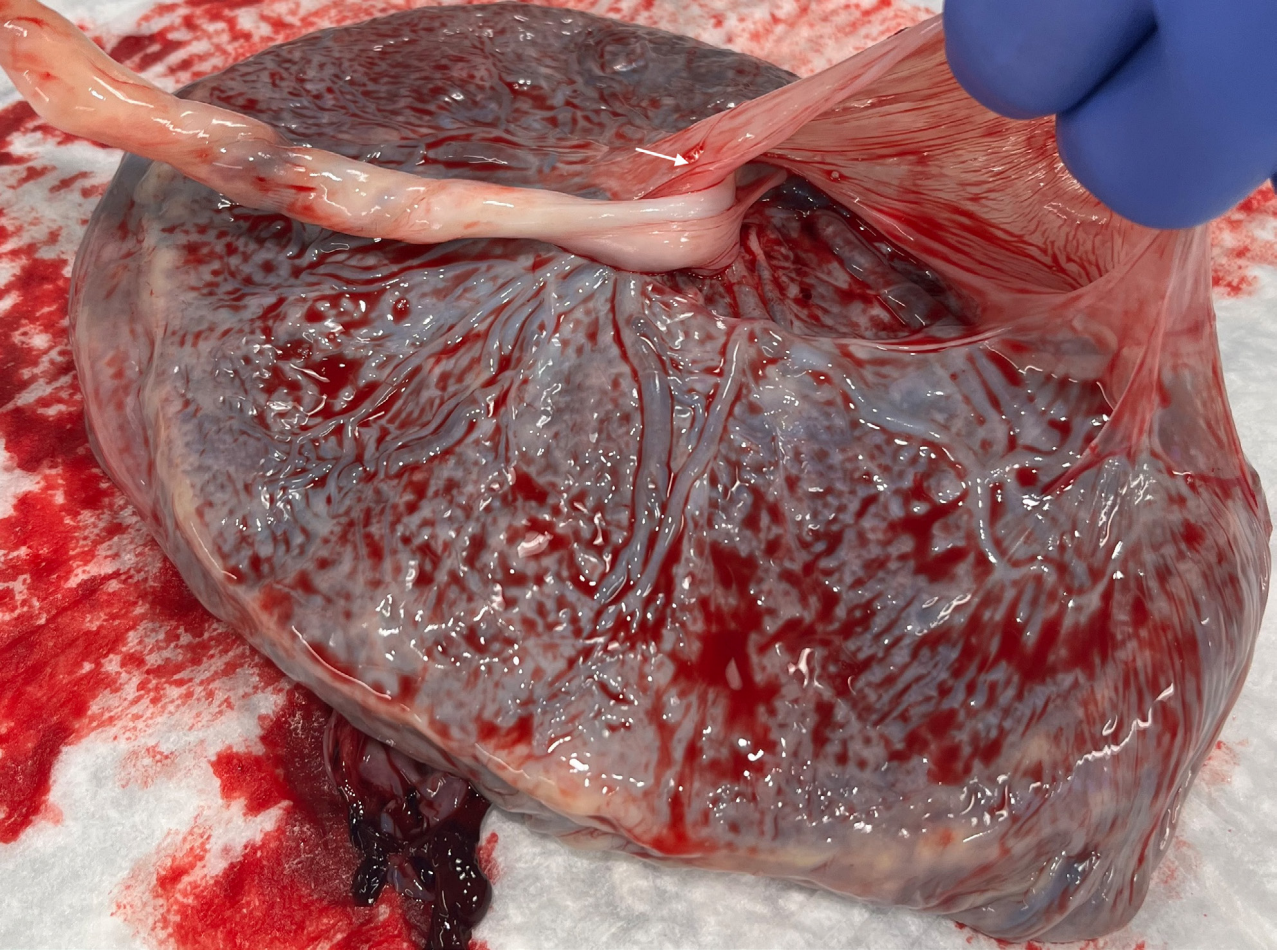
Short umbilical cord with amniotic strands (arrow) on the fetal side of the placenta.

Upon examination of the newborn, we noted truncation of the second through fourth digits of the left hand with connecting bands (Figure 1) and right lower extremity talipes equinovarus (Figure 2). Examination of the placenta revealed a short umbilical cord with amniotic strands on the fetal side of the placenta (Figure 3).

X‐ray of the left hand showed truncation of the second and third proximal and the fourth middle phalanges. X‐ray of the right foot confirmed talipes equinovarus. Genetic testing of the newborn was within normal limits. The patient was discharged at 2 days of life in stable condition with outpatient follow‐up. The patient followed up with orthopedic surgery with management plan to include serial casting of the right clubfoot and plastic surgery of the left hand.

## 2 WHAT IS THE MOST LIKELY DIAGNOSIS?

The diagnosis of amniotic band sequence was made.

## 3 DISCUSSION

Amniotic band sequence is a variable spectrum of congenital anomalies that result from in utero entrapment of fetal parts by amniotic bands. The incidence of amniotic band sequence is estimated to be between 1/1200 and 1/15,000 live births and 1/70 stillbirths.^1^

Prenatal diagnosis of ABS made via fetal ultrasound that detects constriction rings, limb amputation, craniofacial, or body wall defects. Postnatal diagnosis of amniotic band sequence should be suspected in newborns with amniotic bands associated with limb constriction or amputation, body wall defects, and craniofacial abnormalities. Other abnormalities that have also been reported are a short umbilical cord and talipes equinovarus. Examination of fetal side of the placenta will often reveal amniotic strands.^2^

There are no specific guidelines for the management of ABS. Prenatal management of ABS includes fetoscopic in utero lysis of bands. Postnatal management of ABS includes a detailed physical examination and imaging such as x‐ray or ultrasound of the affected body parts to determine the extent of involvement. Genetic testing should be performed, especially if the diagnosis is unclear or genetic syndromes are suspected. Depending on the structures affected an interdisciplinary team including pediatric general surgeon, orthopedic surgeon, and/or plastic surgeon should be involved to develop a treatment plan. The prognosis of ABS depends on the extent of the defects.^3^

## AUTHOR CONTRIBUTIONS

**Jesus Ruiz:** Conceptualization; visualization; writing – original draft.

## CONFLICT OF INTEREST STATEMENT

The author declares no conflict of interest.

## CONSENT

Written informed consent was obtained from the patient to publish this report in accordance with the journal's patient consent policy.

## Data Availability

Data sharing not applicable to this article as no datasets were generated or analysed during the current study
